# Development of QSAR models to predict blood-brain barrier permeability

**DOI:** 10.3389/fphar.2022.1040838

**Published:** 2022-10-20

**Authors:** Sadegh Faramarzi, Marlene T. Kim, Donna A. Volpe, Kevin P. Cross, Suman Chakravarti, Lidiya Stavitskaya

**Affiliations:** ^1^ US Food and Drug Administration, Center for Drug Evaluation and Research, Silver Spring, MD, United States; ^2^ Instem Inc, Columbus, OH, United States; ^3^ Multicase Inc, Beachwood, OH, United States

**Keywords:** blood-brain barrier, permeability, QSAR, *in silico*, log BB

## Abstract

Assessing drug permeability across the blood-brain barrier (BBB) is important when evaluating the abuse potential of new pharmaceuticals as well as developing novel therapeutics that target central nervous system disorders. One of the gold-standard *in vivo* methods for determining BBB permeability is rodent log BB; however, like most *in vivo* methods, it is time-consuming and expensive. In the present study, two statistical-based quantitative structure-activity relationship (QSAR) models were developed to predict BBB permeability of drugs based on their chemical structure. The *in vivo* BBB permeability data were harvested for 921 compounds from publicly available literature, non-proprietary drug approval packages, and University of Washington’s Drug Interaction Database. The cross-validation performance statistics for the BBB models ranged from 82 to 85% in sensitivity and 80–83% in negative predictivity. Additionally, the performance of newly developed models was assessed using an external validation set comprised of 83 chemicals. Overall, performance of individual models ranged from 70 to 75% in sensitivity, 70–72% in negative predictivity, and 78–86% in coverage. The predictive performance was further improved to 93% in coverage by combining predictions across the two software programs. These new models can be rapidly deployed to predict blood brain barrier permeability of pharmaceutical candidates and reduce the use of experimental animals.

## 1 Introduction

The BBB is a primary defense system that protects the brain from exposure to potentially toxic substances and ensures an optimal nutrient supply to the brain. An essential part of BBB is the brain capillary endothelium, a tight membrane junction that separates the blood from the brain tissue and restricts the paracellular transport of compounds across the junction thereby providing selective permeability to the compounds (Abbott et al., 2010). Due to the restrictive nature of the BBB, most compounds enter the brain through either passive diffusion or transporter-mediated uptake. Most hydrophobic compounds pass the BBB through simple diffusion driven by the concentration gradient between the brain and the blood. This process is governed by physiochemical parameters including molecular size, lipophilicity, polar surface area and charge (Begley and Brightman, 2003; Di et al., 2008; Geldenhuys et al., 2015; Copur and Oner, 2017).

In addition to uptake transporters, BBB also hosts efflux transporters that actively transport molecules out of the brain. The most common efflux transporters at the BBB are P-glycoprotein (P-gp, *ABCB1* or Multi-drug resistance 1 (MDR1) protein) and breast cancer resistance protein (BCRP, *ABCG2*) which belong to the family of adenosine triphosphate (ATP) binding cassette (ABC) transporters. Both transporters are often referred to as “gatekeeper” transporters as they provide a vital check on limiting the drugs from accessing the brain (Mahringer and Fricker, 2016). The active uptake transporters are responsible for the uptake of a variety of substrates such as amino acids, fatty acids, essential minerals, vitamins, and glucose. Examples of active transporters include the large neutral amino acid transporter (LAT1) for DOPA and gabapentin. Other uptake transporters relevant for drugs include OATP2A1 and ENT2 (Zamek-Gliszczynski et al., 2018). Active transporters are often targeted to improve the delivery of drugs to the central nervous system (CNS) (Begley and Brightman, 2003; Di et al., 2008; Sanchez-Covarrubias et al., 2014; Geldenhuys et al., 2015; Copur and Oner, 2017).

Investigation of BBB permeability is essential when evaluating the abuse potential of new pharmaceuticals and designing CNS drugs, as only 2% of small molecules cross the BBB (Kola and Landis, 2004; Pardridge, 2005). However, experimental determination of BBB permeability in rodents is often tedious and expensive. As a result, several quantitative structure-activity relationship (QSAR) models have been developed over the years to predict BBB permeation using a variety of methodologies and datasets (Table 1) and to reduce the use of laboratory animals. QSAR models describe the correlation between chemical moieties and their biological activities under the general assumption that similar chemical structures exhibit similar biological activities. QSAR models are particularly useful as they provide rapid, early screening of drugs based upon their chemical structure. Most BBB QSAR models are based on log BB data, which is defined as the logarithmic ratio of the steady-state concentration of a drug in the brain to the blood or plasma. BBB permeability has also been modeled using permeability-surface area (log PS) data and free drug concentration ratio between brain and plasma (K_p,uu,brain_) *in vivo* rodent data (Gratton et al., 1997; Liu et al., 2004; Friden et al., 2009; Loryan et al., 2015; Varadharajan et al., 2015). Although log PS and unbound brain-to-plasma concentration (K_p,uu,brain_) are widely accepted as critical parameters in drug distribution, the publicly available data are limited and therefore the applicability of these models may also be limited (Abraham, 2004; Liu et al., 2004; Friden et al., 2009).

**TABLE 1:** Summary of previously published models and data sets used for log BB prediction.

Study	Method*	Training set (n)	Data source	*R* ^2^**
Young et al. (1988)	MLR	20	*in vivo*	0.69
Van de Waterbeemd and Kansy, (1992a)	MLR	20	*in vivo*	0.70
Abraham et al. (1994)	MLR	57	*in vivo*	0.91
Lombardo et al. (1996)	MLR	55	*in vivo*	0.67
Norinder et al. (1998)	PLS	56	*in vivo*	0.83
Clark, (1999)	MLR	55	*in vivo*	0.77
Luco, (1999)	PLS	58	*in vivo*	0.85
Feher et al. (2000)	PCR	61	*in vivo*	0.73
Keserü and Molnár, (2001)	MLR	55	*in vivo*	0.72
Platts et al. (2001)	MLR	148	*in vivo*, *in vitro*	0.75
Hou and Xu, (2002)	GA	57	*in vivo*	0.88
Doniger et al. (2002)	ANN, SVM	324	*in vivo*, clinical	NA
Ooms et al. (2002)	PLS	83	*in vivo*	0.68
Subramanian and Kitchen, (2003)	LR-PLS	61	*in vivo*, clinical	0.81
Winkler and Burden, (2004)	NN	85	*in vivo*, *in vitro*	0.80
Narayanan and Gunturi, (2005)	VSMP	88	*in vivo*	0.74
Ma et al. (2005)	MLR	37	*in vivo*	0.91
Abraham et al. (2006)	LFER	302	*in vivo*, *in vitro*	0.75
Hemmateenejad et al. (2006)	GA-ANN	123	*in vivo*	NA
Obrezanova et al. (2007)	GP	85	*in vivo*, *in vitro*	0.69
Shen et al. (2008)	GAVS	151	*in vivo*, *in vitro*	0.72
Fu et al. (2008)	MLR	86	*in vivo*, *in vitro*	0.74
Zhang et al. (2008)	*k*NN-Dragon	144	*in vivo*, *in vitro*	0.92
Kortagere et al. (2008)	GRM-SVM	78–376	*in vivo*, *in vitro*, clinical	0.65
Deconinck et al. (2008)	BRT	224	*in vivo*, *in vitro*	0.54
Lanevskij et al. (2009)	Nonlinear regression	125	*in vivo*	0.84
Fan et al. (2010)	GA-MLR	193	*in vivo*	0.72
Muehlbacher et al. (2011)	MLR	362	*in vivo*, *in vitro*	<0.59
Kunwittaya et al. (2013)	SVM	374	*in vivo*, *in vitro*	NA
Yan et al. (2013)	MLR	198	*in vivo*, *in vitro*, clinical	<0.81
Brito-Sanchez et al. (2015)	MLR	381	*in vivo*, *in vitro*	0.69
Wang et al. (2015)	RF	341	*in vivo*, *in vitro*	0.64
Bujak et al. (2015)	MLR	55	*in vivo*	0.84
Zhang et al. (2015)	GA-SVM	260	*in vivo*, *in vitro*	0.67–0.80
Jiang et al. (2016)	SVM	299	*in vivo*, clinical	NA
Dixon et al. (2016)	Kernel-based PLS	644	*in vivo*, *in vitro*	NA
Toropov et al. (2017)	MC-SMILES	250	*in vivo*	0.74
Castillo-Garit et al. (2017)	Classification trees	381	*in vivo*, *in vitro*	NA
Plisson and Piggott, (2019)	ML	300	*in vivo*	NA
Radchenko et al. (2020)	ANN	529	*in vivo*, *in vitro*, clinical	0.81
Singh et al. (2020)	RF, MLP, SMO	432, 479	*in vivo*, *in vitro*	NA
Wu et al. (2021)	ANN	260	*in vivo*, *in vitro*, clinical	0.91
Kim et al. (2021)	ANN	328	*in vivo*, *in vitro*	0.99
Shin et al. (2021)	SVR	153	*in vivo*, *in vitro*	0.64

*MLR: multiple linear regression, PLS: Partial least-squares, PCR: principal component regression, GA: genetic algorithm, NN: neural network, ANN: artificial neural network, SVM: support vector machine, LR: linear regression, VSMP: variable selection and modeling method based on the prediction, LFER: general linear free energy relationship, GP: gaussian processes, GAVS: genetic algorithm based variable selection, *k*NN: *k*-nearest neighbor, GRM: generalized regression model, BRT: boosted regression trees, RF: random forest, MC: monte carlo, SMILES: Simplified molecular input-line entry systems, ML: machine learning, MLP: multilayer perceptron, SMO: sequential minimal optimization, SVR: support vector regression. **NA: not applicable.

There are several molecular descriptors that have been used to predict BBB permeability including lipophilicity, polar surface area, and hydrogen bonding ability (Young et al., 1988; Van de Waterbeemd and Kansy, 1992a; Abraham et al., 1994; Clark, 1999). However, more recently, 2D structure-based dragon descriptors (Zhang et al., 2008), 3D structure-based VolSurf descriptors (Crivori et al., 2000), solvation free energies (Lombardo et al., 1996), and 3D conformations (Keserü and Molnár, 2001) have been used in making BBB models. Additionally, in the earlier studies, multiple linear regression (MLR) analysis was utilized to relate molecular descriptors to log BB. One shortcoming of the MLR analysis is the finite number of descriptors that could be employed. Other methods that have been employed include partial least square analysis, genetic algorithms (GA), random forest (RF), support vector machine (SVM) and artificial neural networks (ANN).

A common limitation among many of the previously constructed models is their small training set size, which limits their applicability in a regulatory environment. Although numerous models have been developed in the last decade using much larger training sets (n = 1,000+), these datasets often contain a combination of data types including *in silico* predicted data, experimental data from *in vitro* and *in vivo* studies, and clinical side effects data (Martins et al., 2012; Gao et al., 2017; Fan et al., 2018; Wang et al., 2018; Yuan et al., 2018; Miao et al., 2019; Alsenan et al., 2020, 2021; Liu et al., 2021). Other limitations of the data sets used in previous QSAR models include (i) the use of indirect measurements, (ii) use of unverified or wrongly interpreted data, and (iii) lack of chemical diversity. Finally, challenges affecting implementation of previously developed models such as updating training set data limit the applicability of those models (Fan et al., 2010).

In the present study, two statistical-based models for predicting BBB permeability have been constructed using Leadscope Enterprise (*LS*) and CASE Ultra (*CU*). The new training sets contain *in vivo* rodent data from drugs, drug metabolites and non-drugs, and have the largest number of chemicals compared to previously published models trained on *in vivo* data. Moreover, the quality of the underlying training data has been enhanced through careful review of original experiments to resolve or remove discrepant studies. In addition, predictive performance of the newly constructed models has been assessed using both internal and external validation experiments and showed good predictive accuracy. Finally, these new models can be rapidly used to design CNS drugs and to assess abuse potential of drug candidates.

## 2 Methods

### 2.1 Data sources

All training set data used to construct BBB permeability model were comprised of non-proprietary data harvested from published literature (*e.g*., PubMed, Web of Science v.5.34, Scopus, Elsevier, and Google Scholar), US FDA approval packages (*e.g*., Drugs@FDA and PharmaPendium^®^), EMA approval packages (*e.g*., PharmaPendium^®^), and patents. All references for BBB databases are provided in Supplementary Table S1.

### 2.2 Data scoring

The BBB permeability database contains blood/plasma (B/P) or blood/brain (B/B) ratios obtained from rodents that were treated *via* intravenous, intraperitoneal, or oral routes. For the majority of data entries, the amount of the chemical present in the brain and blood or plasma was measured in the animals 30 min to a several hours after administration. However, in some cases, the animals were sacrificed at certain intervals after treatment and different B/P ratios were reported. In such cases, the ratio of the area under the curve (AUC) for the brain and plasma concentrations were used. In experiments where different amounts of a chemical were reported in different parts of the brain, the average value was considered. All findings were transformed into a binary scoring system for modeling purposes, where “0” denotes a negative finding (no brain penetration) and “1” denotes a positive finding (brain penetration). Chemicals with a log BB ≥ -1 were considered positive while chemicals with a log BB < -1 were considered negative (Vilar et al., 2010). The final BBB database is comprised of 921 compounds with 52% positives. The dataset and references are provided in Supplementary Table S1.

### 2.3 Chemical structure curation

The chemical structures were obtained from SciFinder^®^ and published literature. Electronic representations of chemical structures were created using structure data file (SDF) format. Inorganic chemicals, noble gases, mixtures, single atoms, metals, and high molecular weight compounds (MW ≥ 1800; polysaccharides, proteins, polymers, *etc*.) were excluded from the training set due to processing limitations within the QSAR software. Furthermore, the neutralized free form of any simple salt was included. A final manual inspection was performed to ensure the chemicals, their associated data and references were accurately recorded.

### 2.4 QSAR software

Two commercial QSAR software platforms, *Leadscope Enterprise (LS)* version 3.9 (Instem Inc., United States), and CASE Ultra (CU) version 1.8.0.1 (MultiCASE Inc., United States) were used to construct two distinct binary QSAR models. All software programs were acquired and used under Research Collaboration Agreements between FDA/CDER and the software providers mentioned above.

#### 2.4.1 Leadscope Enterprise (LS)

*LS* is a data mining, visualization, and advanced informatics application that includes the capability to build and apply QSAR models. To construct QSAR models for BBB, a training set of 921 chemicals was imported into *LS* and fingerprinted using a set of 27,142 pre-defined medicinal chemistry structural features as candidate descriptors for model building. A small predictive subset of these features was used to construct the model. Additionally, a set of unique scaffolds was automatically constructed from the pre-defined structural features that specifically defined structure-activity relationships in the training set. The unique set of scaffolds was generated for the BBB permeability model using the following settings: 1) a minimum of three compounds per scaffold; 2) a minimum six of atoms per scaffold; 3) no restriction on the maximum number of rotatable bonds; and 4) a minimum absolute Z-score of 1.0. Z-score of a structural features is the difference between the mean activity of the subset of compounds having that feature and the mean activity of the full set (Roberts et al., 2000). Molecular properties such as molecular weight, number of rotatable bonds, number of hydrogen bond donors, number of hydrogen bond acceptors, Lipinski score, AlogP (logarithm of 1-octanol/water partition coefficient), polar surface area, and atom count were calculated using Leadscope. The squared Pearson correlation coefficients (*R* ^2^) for the molecular properties were computed using python and added to the models to improve predictive performance.

Highly predictive features and the corresponding helper features were identified in the feature editor for retention while weakly predicted features were removed using Z-score, frequency, precision and mean activity as discriminating parameters (Roberts et al., 2000). Subsequently some features were divided to better define their chemical environment (acyclic vs cyclic) or expanded using the expand features to more specifically define their functional groups. Additional pruning was manually performed to reduce the number of features while maintaining optimal predictive performance. Specifically, redundant features, highly overlapping or similar features, and coincidental features that were highly correlated were removed. Lastly, the total number of model features was reduced using a partial least-squared regression algorithm leaving only those that best fit the experimental activity scores in the training set (Roberts et al., 2000).

For BBB model, cross-validation was performed 10 times using a 10 × 10% leave-many-out (LMO) method. This method randomly selects 10% of the training set for testing and reconstructs a reduced model using the remaining 90% of the compounds and recalculates the descriptor weights. This process was repeated 10 times with 10 diverse training sets ensuring that all the compounds present in the training set were predicted ten times. The average predicted values were used in calculating the Cooper statistics (Cooper et al., 1979).

A classification threshold was determined by varying the positive cutoff probability thresholds for equivocal results and analyzing the resulting Cooper statistics. The optimal probability range for indeterminate predictions for the BBB model were identified to be 0.4 to 0.6. Predictions that are above the 0.6 probability cutoff were classified as positive, while predictions below 0.4 were classified as negative. A chemical was treated as out-of-domain (OOD) in instances where the test chemical did not contain any structural model features or showed a lack of similarity to the training set compounds (at least 30% similarity to a single training set compound is required).

#### 2.4.2 CASE Ultra (CU)

*CU* is a QSAR software platform that builds models using various machine learning algorithms applied on training sets of chemical structures and their activity labels. The algorithm automatically generates molecular fragments from the training structures and uses them as descriptors. A CU model contains a set of structural alerts and deactivating features identified from the training data. The structural alerts are substructures primarily associated with active training compounds and the deactivating features decrease the potency of the alerts. These features are incorporated in a global logistic regression QSAR model and therefore contains positive and negative quantitative weights. During application of the model, the alerts and deactivating features are searched in the test chemical, and the regression model is used to generate a score between 0 and one to indicate the likelihood of the test chemical being positive. The model also verifies if all three-atom linear fragments generated from the test compounds are present in the training structures to establish that the test chemical is within the applicability domain of the model. No hyper-parameter optimization is performed.

The BBB model was constructed in *CU* using a training set of 921 chemicals. The models were cross-validated internally 10 times using a previously described 10 by 10% LMO method. The classification threshold was selected based on optimal balance between sensitivity and specificity on the receiver operating characteristic (ROC) curve. During model application, predictions were classified as equivocal when a predicted confidence was within ±0.1 of the classification threshold. Predicted values above the upper bound of this range were treated as positive, and those below this range were treated as negative. An out-of-domain (OOD) response was given to any chemicals that contained one or more unknown fragments not recognized by the model and do not contain combination of alerts/features strong enough to give a positive prediction.

### 2.5 External validation

The predictive performance of the BBB models was assessed using an external validation set comprised of 83 chemicals (42 positives and 41 negatives) obtained from published literature. All references and activity scores are provided in Supplementary Table S2 for the external validation set.

### 2.6 Combining model outputs in external validation

To examine the combined predictive performance of *LS* and *CU*, a positive prediction from any one software platform was used to justify an overall positive prediction. Similarly, an equivocal prediction from any one software platform was used to justify an overall equivocal prediction, in the absence of a positive prediction. In the case that one of the models was OOD and the other model generated a prediction, the OOD was disregarded and the prediction was used to generate an overall call. An overall negative prediction was reported when a statistical model generated a negative prediction in the absence of positive or equivocal predictions from the other model.

### 2.7 Performance statistics

In order to evaluate the performance of individual model outputs, Cooper statistics was employed. Briefly, predictive performance was evaluated using a classic 2x2 contingency table containing counts of true positives (TP), true negatives (TN), false positives (FP), and false negatives (FN). Chemicals classified as OOD and equivocal were excluded from Cooper statistic calculations. Statistics such as sensitivity [TP/(TP + FN)], specificity [TN/(TN + FP)], positive predictivity [TP/(TP + FP)], negative predictivity [TN/(TN-FN)], and accuracy [(TP + TN)/(TP + TN + FP + FN)] were calculated as described by Cooper et al., 1979 (Cooper et al., 1979). Coverage was calculated as the percentage of all chemicals screened for which a prediction could be made (OOD results do not constitute a prediction).

## 3 Results

### 3.1 Database overview

**FIGURE 1:**
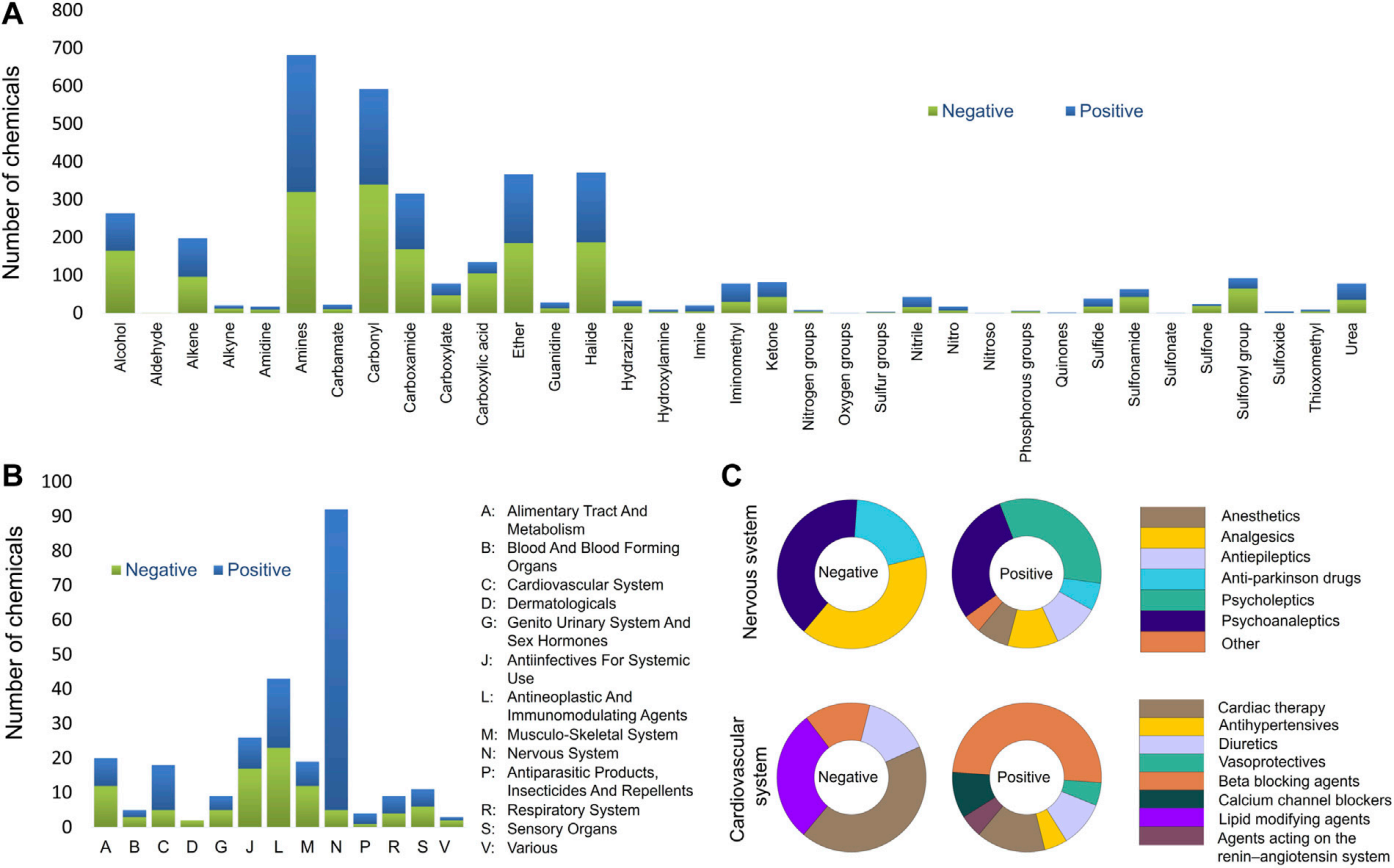
Database analysis **(A)** Assessment of the functional groups present in the entire BBB database. **(B)** Anatomical Therapeutic Chemical (ATC) level 1 classes present BBB database. **(C)** ACT level 2 classes of the nervous and cardiovascular systems.

**FIGURE 2:**
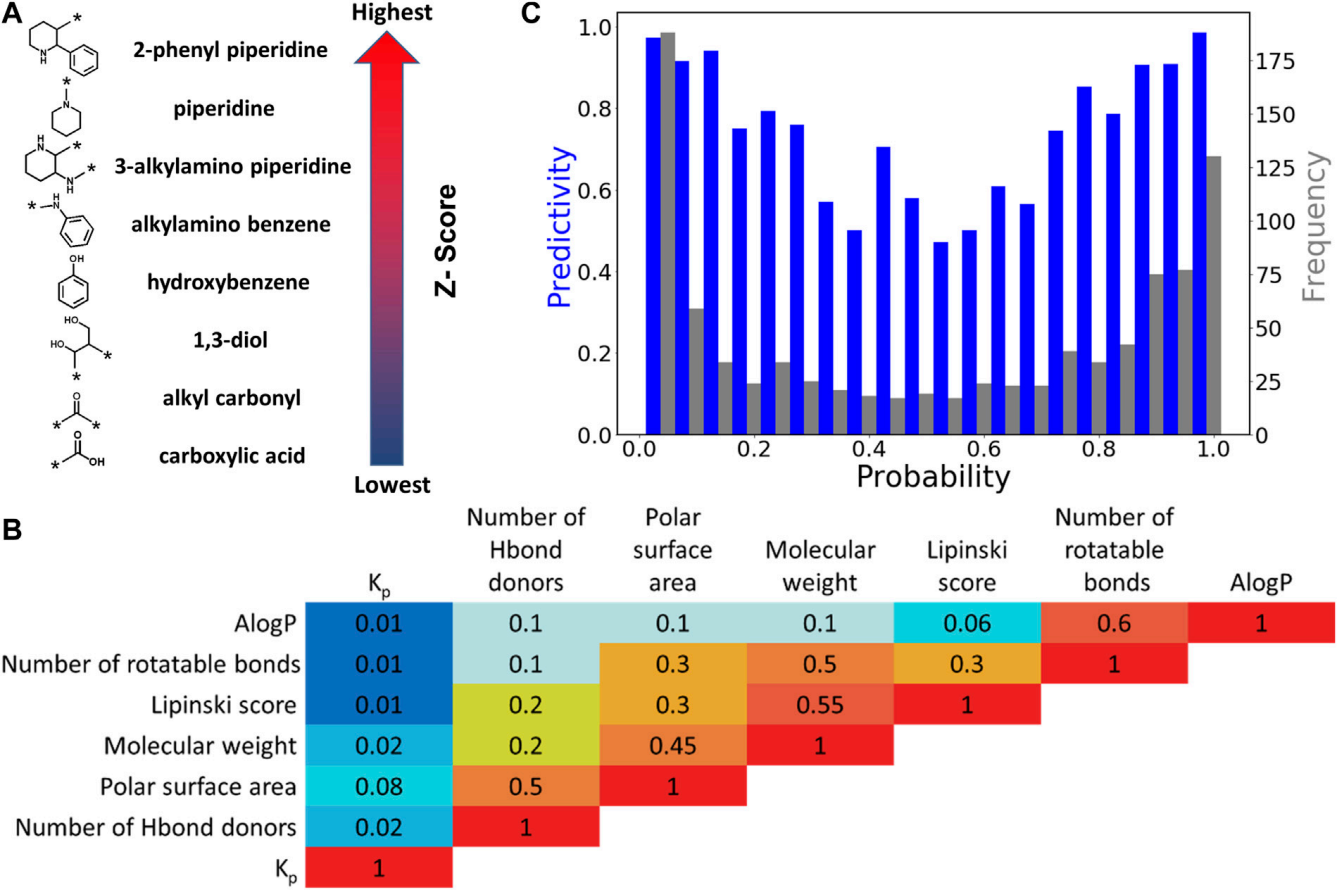
Leadscope model analysis. **(A)** Selected chemical features with highest and lowest Z-scores. The arrow shows the order of Z-scores associated with features in the model. **(B)** Correlation (*R* ^2^) between pairs of physicochemical features. **(C)** Histogram of the predictivity (blue bars) and frequency (grey bars) as a function of probability in *LS* model.

In the present study, the rodent BBB permeability dataset was compiled from publicly available data sources and the original study data were used. The results from rats and mice were treated as equivalent since previous studies show no significant difference in brain permeability between rats and mice (Murakami et al., 2000; Abraham et al., 2006). The final BBB permeability database contains 921 unique chemicals of which 621 compounds are from *in vivo* studies in rats and 300 in mice. The database is well-balanced with a total of 478 compounds scored as positive and 443 as negative (activity scores provided in Supplementary Table S1). Furthermore, the database is comprised of 263 drug substances approved between 1939 and 2022, 21 drug derivatives, 13 drug metabolites, 61 investigational drugs undergoing clinical trials, 14 prodrugs, and 549 other non-drug molecules. This database covers a broad range of chemical space, functional groups, and Anatomical Therapeutic Chemical (ATC) classes as presented in Figure 1. Most functional groups and ATC classes have an equal distribution between positives and negatives in the database. Chemicals that contain carboxylic acid, sulfone, sulfonyl and sulfonamide functional groups were mostly negative. As expected, the majority of central nervous system drugs in the database cross the BBB. However, two triptan analgesics (rizatriptan and almotriptan) were identified among negative drugs (Figure 2B). A possible reason is that triptans are usually substrates of human, but not rat, BBB uptake transporters (Zhang et al., 2016). Another interesting finding is that majority of the cardiovascular drugs in the database cross the BBB. A review of the literature suggested that the lipophilicity of many cardiovascular drugs, specifically beta blocking agents, may be the reason for their BBB permeability (McAinsh and Cruickshank, 1990; Goldner, 2012; Shah et al., 2020). When compared to several of the previously described models, the current training set showed improved coverage of almost all chemical functional groups (Supplementary Table S5).

### 3.2 QSAR model development

Previous modeling efforts employed calculated physicochemical descriptors such as polar surface area (PSA), number of hydrogen donors/acceptors, and molecular weight to predict BBB (Young et al., 1988; van de Waterbeemd and Kansy, 1992b; Abraham et al., 1994; Lombardo et al., 1996; Norinder et al., 1998; Clark, 1999; Luco, 1999; Feher et al., 2000; Keserü and Molnár, 2001; Platts et al., 2001; Abraham, 2004; Abraham et al., 2006). While these properties influence BBB permeability of molecules and can be applied to simple cases, they are limited in their ability to comprehensively predict BBB permeability of drugs that pass through more complex mechanisms. In the present study, machine-learning algorithms were used to examine all structural features present in the training set and global molecular properties that are useful to predict and interpret BBB permeability. The two modeling platforms that were used to construct BBB models are *LS* and *CU*.

The *LS* QSAR model was optimized by manual refinement of chemical structural features and physicochemical descriptors. Highly predictive features were identified for retention while 14 redundant and less discriminating chemical features were removed. The total number of chemical features present in the final BBB model is 386. Examples of chemical features with highest and lowest Z-scores, corresponding to highest and lowest BBB permeability are presented in Figure 2A. Chemical features with highest Z-scores are comprised of aliphatic and aromatic rings while carboxylic acids and carbonyls have the lowest Z-scores. Moreover, polycyclic secondary and tertiary amines are also positive features.

Additionally, six physicochemical descriptors including molecular weight, number of rotatable bonds, number of hydrogen bond donors, Lipinski score, AlogP, and PSA were assessed for their predictive ability. The overall results showed a very poor correlation between the individual physicochemical descriptors and log BB alone. Specifically, the squared Pearson correlation coefficient (*R* ^2^) values for log BB ratio and molecular weight, PSA, and number of hydrogen bond donors are 0.02, 0.08 and 0.02, respectively (see Figure 2B and Supplementary Figure S1). However, it should be noted that a 3% increase in statistical performance was observed upon inclusion of the six molecular descriptors. The predictivity of the model and frequency of the compounds as a function of probability is presented in Figure 2C. The U-shaped plots indicate the optimum regression, with the maximum probability located at the two ends of the axis. The lowest predictivity and frequency was identified to be at approximately 0.5 and selected as the equivocal range.

**FIGURE 3:**
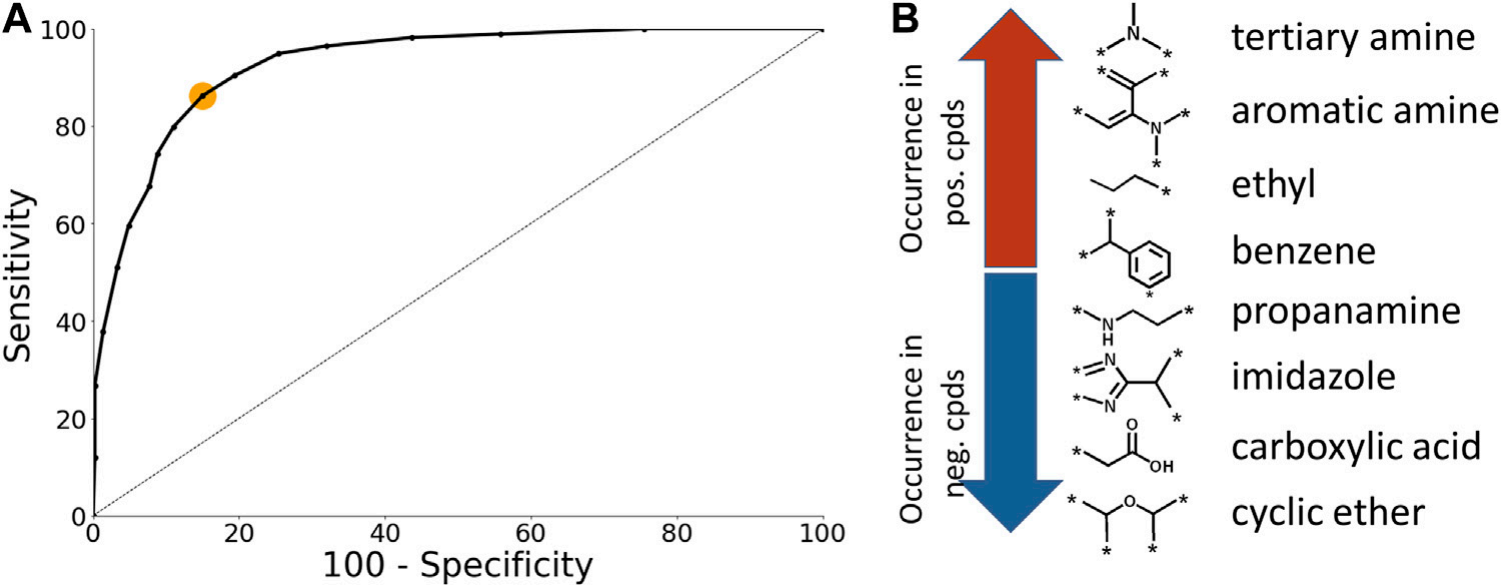
Case Ultra model analysis. **(A)** ROC plot of the BBB model. The orange dot corresponds to the optimal classification threshold **(B)** Selected examples of chemical fragments with highest number of positive and negative compounds.

The *CU* models were optimized using ROC plots that were generated by varying the classification threshold which defines a positive prediction (Figure 3A). The optimal classification threshold was identified to be 0.55 (Figure 3A; orange dot). The number of chemical fragments present in the *CU* model is 171. Selected examples of chemical fragments with the highest number of positive and negative compounds are presented in Figure 3B. Chemical fragments with the highest number of chemicals that permeate the BBB contain aromatic moieties and amines while chemical fragments with the highest number of negative chemicals contain carboxylic acids and cyclic ethers.

### 3.3 Performance statistics of BBB permeability model using cross-validation and external validation

The predictive performance statistics for the BBB models based on 10% LMO cross-validation experiments as well as the external validation experiments are presented in Table 2. However, it should be noted that the coverage in the cross-validation statistics from *LS* and *CU* cannot be compared directly as they are calculated differently. The *CU* coverage is calculated using domain analysis while *LS* provides a prediction for all the chemicals in the cross-validation experiment. The *LS* model achieved a sensitivity of 82% and a negative predictivity of 80% in cross-validation, while the *CU* model achieved a sensitivity of 85% and a negative predictivity of 83%. Furthermore, when using an external validation set of 83 chemicals (51% positive; 28 drugs and 55 non-drug molecules), the *LS* model achieved a sensitivity of 70% and a negative predictivity of 72%, while the *CU* model achieved a sensitivity of 75% and a negative predictivity of 70%. The partitioned predictive performance for drugs and nondrugs is provided in Supplementary Table S3. Additionally, a prediction comparison analysis for *LS* and *CU* by functional groups and drug classes is provided in Supplementary Figures S2–4.

**TABLE 2:** Validation statistics for BBB permeability QSAR models. Columns 2 and 3 show cross-validation performance statistics and columns 4–6 show external validation performance statistics for single and combined models.

Statistics	Cross-validation (10% LMO)		External validation (n = 83; 51% pos)		
Software platforms	*LS*	*CU*	*LS*	*CU*	*LS/CU*
Sensitivity	82%	85%	70%	75%	80%
Specificity	78%	86%	68%	72%	51%
Positive Predictivity	80%	88%	66%	77%	64%
Negative Predictivity	80%	83%	72%	70%	70%
Accuracy	80%	86%	69%	74%	66%
Coverage	100%	70%	86%	78%	93%
Chi-squared	336	35	10.3	14	8
Matthews Correlation Coefficient	0.6	0.71	0.38	0.6	0.4

In a subsequent evaluation, the combined predictive performance of the *LS* and *CU* models was assessed (Table 2). Here, the models achieved a sensitivity of 80% and negative predictivity of 70%. However, a decrease in specificity (51%) and positive predictivity (64%) was observed when predictions across the two software programs were combined. These results were anticipated given that combining predictions across different software platforms results in an increase of false positive predictions. A total of 11 chemicals were outside the applicability domain for *LS* while 18 were outside applicability domain for *CU*. However, when predictions from the *LS* and *CU* were combined, 93% of all chemicals were within the applicability domain.

## 4 Discussion

### 4.1 Database development

Obtaining meaningful alerts and a robust QSAR model depend heavily on the quality of the training set data. In the present study, efforts were made to identify and extract high quality data for the BBB permeability model. One of the most commonly reported and trusted measures for BBB permeability is log BB; this parameter is generated by most pharmaceutical companies for drug candidates. Among the challenges of combining Log BB data from multiple sources is the potential for introducing conflicting data into models thereby affecting the quality of the data. To enhance the overall quality of the underlying data, chemicals with contradictory and/or equivocal study results were reviewed and resolved or removed from the databases.

Recently, several studies have suggested that the steady state unbound brain-to-plasma ratio, K_p,uu,brain_ is a relevant parameter to measure drug concentration as the key driving force for drug distribution is the free concentration in the brain. However, publicly available data for K_p,uu,brain_ are very limited and therefore a viable model to predict K_p,uu,brain_ could not be developed at this time.

### 4.2 Role of descriptors in BBB permeability

Previously published models employed calculated physicochemical descriptors such as lipophilicity, PSA and/or hydrogen bonding (Young et al., 1988; Van de Waterbeemd and Kansy, 1992a; Abraham et al., 1994; Clark, 1999). There is a general agreement that these specific descriptors can influence log BB (Clark, 2003). For instance, lipophilicity is positively correlated with log BB (Young et al., 1988; Calder and Ganellin, 1994; Kaliszan and Markuszewski, 1996; Salminen et al., 1997; Goodwin and Clark, 2005) while hydrogen bonding is negatively correlated to brain penetration (Van de Waterbeemd and Kansy, 1992a; Calder and Ganellin, 1994; Clark, 1999). In addition, several reports indicate log BB is negatively correlated to molecular weight (Calder and Ganellin, 1994; Kaliszan and Markuszewski, 1996; Salminen et al., 1997; Kaznessis et al., 2001; Platts et al., 2001). In this investigation, the use physicochemical descriptors was found to improve the overall performance of the *LS* models (by 3%) when combined with chemical features, although physicochemical descriptors were poorly correlated with experimental log BB parameter alone. This can be attributed to the larger log BB data set that covers a more diverse chemical space (Brito-Sanchez et al., 2015). It is anticipated that as more data become available, finding a single equation that describes log BB as a function of physicochemical descriptors will become more difficult. Therefore, models that use a combination of chemical and physicochemical features may be advantageous.

A review of alerts and deactivating chemical features in *LS* and *CU* models revealed that the top features with highest activity scores belong to polycyclic aromatic compounds. The training set structures representing these alerts are relatively nonpolar (lipophilic), which is favorable for crossing the BBB. In addition, unlike primary amines, polycyclic secondary and tertiary amines are among the top positive alerts. Beside the reduced polarity of those amines, the ability of making hydrogen bonds is also reduced compared to primary amines, which may explain their higher BBB permeability (Silverman et al., 2009). In contrast, features that contained carboxylic acids and alcohols had the lowest activity scores presumably due to their ability to form hydrogen bonds (Abraham et al., 1994). At physiological pH of 7.4, carboxylic acids are dissociated to carboxylate ions, which improves their water solubility and the ability to form hydrogen bonds (Bredael et al., 2022). The low lipophilicity of carboxylic acids, at physiological pH, also limits their BBB penetration (Soloway et al., 1960). Additionally, ethers were also found among negative features due to their ability to accept hydrogen bonds. However, one should be aware of exceptions to the hydrogen bond rule. As discussed earlier, there is a low correlation between log BB and number of hydrogen bond acceptors/donors. A detailed assessment of the functional groups that are present in the BBB database showed that the current training set contains 135 compounds that have carboxylic acid groups with 30 being BBB permeable (Figure 1A). Compounds with carboxylic acids that pass BBB are typically substrates of uptake transporters. An example of this is l-DOPA, a precursor for dopamine, which has a carboxylic acid and a diol group and is capable of crossing BBB (Di et al., 2013).

### 4.3 External validation of BBB QSAR models

In this investigation, an external validation set was used to examine BBB models individually and by combining predictions across *LS* and *CU*. In a regulatory setting, high sensitivity and negative predictivity are preferred to reduce the risk of false negatives and minimize the risk to public health. Towards this end, the current BBB models were tuned to achieve high sensitivity and negative predictivity while maintaining good overall predictive performance in other statistical parameters. Specifically, the new models showed a sensitivity ranging from 70 to 75% and negative predictivity ranging from 70 to 72% in external validation. Furthermore, when predictions from the two methodologies were combined, a sensitivity of 80% and coverage of 93% was achieved. While the increase in the false positive rate is not ideal when predictions are combined, it can be mitigated by evaluating the alerts behind the positive prediction and examining structurally similar analogs. Perhaps the most striking finding was that OOD chemicals in *CU* were successfully predicted by *LS,* suggesting that the two software platforms interpret chemicals differently resulting in different OOD domain predictions. Moreover, the overall increase in coverage is desirable for predicting a large diversity of chemicals.

BBB penetration of drugs is a complicated process involving passive diffusion and active transport (efflux or uptake). The current data set includes known substrates of active transporters. The model is agnostic to such complicated processes. Furthermore, the log BB data entries are collected from different experiments where drugs are administered through different routes and brain samples are collected at various time points post administration. Despite all these complications, the model can estimate BBB permeation with relatively high precision. In future, utilization of a combination of models for different transport mechanisms may further improve the log BBB predictivity.

## 5 Conclusion

In the present study, a complementary computational model has been developed using two software platforms, *LS* and *CU* to predict whether an unknown substance can penetrate the blood-brain barrier (BBB). The model has a large training set and includes up-to-date information for drugs and their metabolites, and non-drugs to provide an optimal domain of applicability. Advantages of the current data set over previous ones are (i) exclusive use of data from *in vivo* rodent experiments and (ii) use of a more balanced dataset, which allows for more accurate modeling. The current models demonstrate improved coverage of chemical functional groups over several of the previously described models and show good sensitivity and negative predictivity, which are critical parameters for the safety assessment. Furthermore, the use of two software platforms was found to increase coverage to 93%. When predictions are in consensus, greater confidence can be inferred. However, when predictions are inconclusive or conflicting among the two software platforms, an expert review can provide supporting information.

In conclusion, the newly constructed models can be rapidly deployed during drug development to predict BBB permeability of drugs and their metabolites and reduce the need to test laboratory animals. Identification of drug candidates that cross the BBB can inform strategies for derisking the potential for abuse liability and to assist with designing CNS drugs.

## Data Availability

The original contributions presented in the study are included in the article/Supplementary Material further inquiries can be directed to the corresponding author.

## Glossary

ABC: ATP binding cassette transporters
ANN: Artificial neural networks
ATP: Adenosine triphosphate
B/P: Brain concentration/plasma concentration
BB: Brain concentration/blood concentration
BBB: Blood-brain barrier
BCRP: Breast cancer resistant protein
BRT: Boosted regression trees
CDER: Center for drug evaluation and research
CU: CASE Ultra
DT: Decision tree
FN: False negatives
FP: False positives
GA: Genetic algorithm
GA-CG-SVM: Genetic algorithm-conjugate gradient-SVM
GAVS: Genetic algorithm based variable selection
*k*NN: *k*-nearest neighbor
K_p,uu,brain_: Unbound brain-to-plasma concentration
LDA: Linear discriminant analysis
LLC-PK1: Lilly Laboratories cell-porcine kidney cells
LMO: Leave-many-out
LS: Leadscope
MC: Monte Carlo
MDR1: Multi-drug resistance protein 1 (same as P-gp)
ML: Machine learning
MLP: Multilayer perceptron
MLR: Multiple linear regression
NA: not applicable
NN: Neural network
OOD: Out-of-domain
PCR: Principle component regression
P-gp: P-glycoprotein
PHASE: Public health assessment *via* structural evaluation
PLS: Partial least-squares
PS: Permeability-surface area
PSA: Polar surface area
QSAR: Quantitative structure-activity relationship
RF: Random forest
ROC: Receiver operating characteristic
SMILES: Simplified molecular input-line entry systems
SMO: Sequential minimal optimization
SVM: Support vector machine
SVR: Support vector regression
TN: True negatives
TP: True positives
VSMP: Variable selection and modeling method based on the prediction

## References

[B1] Abbott N. J. Patabendige A. A. Dolman D. E. Yusof S. R. Begley D. J. (2010). Structure and function of the blood–brain barrier. Neurobiol. Dis. 37, 13–25. 10.1016/j.nbd.2009.07.030 19664713

[B2] Abraham M. H. Chadha H. S. Mitchell R. C. (1994). Hydrogen bonding. 33. Factors that influence the distribution of solutes between blood and brain. J. Pharm. Sci. 83, 1257–1268. 10.1002/jps.2600830915 7830242

[B3] Abraham M. H. Ibrahim A. Zhao Y. Acree W. E. (2006). A data base for partition of volatile organic compounds and drugs from blood/plasma/serum to brain, and an LFER analysis of the data. J. Pharm. Sci. 95, 2091–2100. 10.1002/jps.20595 16886177

[B4] Abraham M. H. (2004). The factors that influence permeation across the blood-brain barrier. Eur. J. Med. Chem. 39, 235–240. 10.1016/j.ejmech.2003.12.004 15051171

[B5] Alsenan S. Al-Turaiki I. Hafez A. (2021). A deep learning approach to predict blood-brain barrier permeability. PeerJ. Comput. Sci. 7, e515. 10.7717/peerj-cs.515 34179448 PMC8205267

[B6] Alsenan S. Al-Turaiki I. Hafez A. (2020). A recurrent neural network model to predict blood–brain barrier permeability. Comput. Biol. Chem. 89, 107377. 10.1016/j.compbiolchem.2020.107377 33010784

[B7] Begley D. J. Brightman M. W. (2003). “Structural and functional aspects of the blood-brain barrier,” in Peptide transport and delivery into the central nervous system. Editors Prokai L. Prokai-Tatrai K. (Basel: Birkhäuser Basel), 39–78. 10.1007/978-3-0348-8049-7_2 14674608

[B8] Bredael K. Geurs S. Clarisse D. De Bosscher K. D’hooghe M. (2022). Carboxylic acid bioisosteres in medicinal chemistry: Synthesis and properties. J. Chem. 2022, 1–21. 10.1155/2022/2164558

[B9] Brito-Sanchez Y. Marrero-Ponce Y. Barigye S. J. Yaber-Goenaga I. Morell Perez C. Le-Thi-Thu H. (2015). Towards better BBB passage prediction using an extensive and curated data set. Mol. Inf. 34, 308–330. 10.1002/minf.201400118 27490276

[B10] Bujak R. Struck-Lewicka W. Kaliszan M. Kaliszan R. Markuszewski M. J. (2015). Blood–brain barrier permeability mechanisms in view of quantitative structure–activity relationships (QSAR). J. Pharm. Biomed. Anal. 108, 29–37. 10.1016/j.jpba.2015.01.046 25703237

[B11] Calder J. A. Ganellin C. R. (1994). Predicting the brain-penetrating capability of histaminergic compounds. Drug Des. Discov. 11, 259–268. 7727679

[B12] Castillo-Garit J. A. Casanola-Martin G. M. Le-Thi-Thu H. Barigye S. J. (2017). A simple method to predict blood-brain barrier permeability of drug-like compounds using classification trees. Med. Chem. 13, 664–669. 10.2174/1573406413666170209124302 28185535

[B13] Clark D. E. (2003). *In silico* prediction of blood-brain barrier permeation. Drug Discov. Today 8, 927–933. 10.1016/s1359-6446(03)02827-7 14554156

[B14] Clark D. E. (1999). Rapid calculation of polar molecular surface area and its application to the prediction of transport phenomena. 2. Prediction of blood-brain barrier penetration. J. Pharm. Sci. 88, 815–821. 10.1021/js980402t 10430548

[B15] Cooper J. A. 2nd Saracci R. Cole P. (1979). Describing the validity of carcinogen screening tests. Br. J. Cancer 39, 87–89. 10.1038/bjc.1979.10 758931 PMC2009815

[B16] Copur T. Oner L. (2017). “Drug delivery to the brain: Pharmacokinetic concepts,” in Nanotechnology methods for neurological diseases and brain tumors (Elsevier), 69–89.

[B17] Crivori P. Cruciani G. Carrupt P.-A. Testa B. (2000). Predicting Blood−Brain barrier permeation from three-dimensional molecular structure. J. Med. Chem. 43, 2204–2216. 10.1021/jm990968+ 10841799

[B18] Deconinck E. Zhang M. H. Petitet F. Dubus E. Ijjaali I. Coomans D. (2008). Boosted regression trees, multivariate adaptive regression splines and their two-step combinations with multiple linear regression or partial least squares to predict blood-brain barrier passage: A case study. Anal. Chim. Acta 609, 13–23. 10.1016/j.aca.2007.12.033 18243869

[B19] Di L. Kerns E. H. Carter G. T. (2008). Strategies to assess blood–brain barrier penetration. Expert Opin. Drug Discov. 3, 677–687. 10.1517/17460441.3.6.677 23506148

[B20] Di L. Rong H. Feng B. (2013). Demystifying brain penetration in central nervous system drug discovery: Miniperspective. J. Med. Chem. 56, 2–12. 10.1021/jm301297f 23075026

[B21] Dixon S. L. Duan J. Smith E. Von Bargen C. D. Sherman W. Repasky M. P. (2016). AutoQSAR: An automated machine learning tool for best-practice quantitative structure–activity relationship modeling. Future Med. Chem. 8, 1825–1839. 10.4155/fmc-2016-0093 27643715

[B22] Doniger S. Hofmann T. Yeh J. (2002). Predicting CNS permeability of drug molecules: Comparison of neural network and support vector machine algorithms. J. Comput. Biol. 9, 849–864. 10.1089/10665270260518317 12614551

[B23] Fan J. Yang J. Jiang Z. (2018). Prediction of central nervous system side effects through drug permeability to blood–brain barrier and recommendation algorithm. J. Comput. Biol. 25, 435–443. 10.1089/cmb.2017.0149 29058464

[B24] Fan Y. Unwalla R. Denny R. A. Di L. Kerns E. H. Diller D. J. (2010). Insights for predicting blood-brain barrier penetration of CNS targeted molecules using QSPR approaches. J. Chem. Inf. Model. 50, 1123–1133. 10.1021/ci900384c 20578728

[B25] Feher M. Sourial E. Schmidt J. M. (2000). A simple model for the prediction of blood–brain partitioning. Int. J. Pharm. 201, 239–247. 10.1016/s0378-5173(00)00422-1 10878329

[B26] Friden M. Winiwarter S. Jerndal G. Bengtsson O. Wan H. Bredberg U. (2009). Structure-brain exposure relationships in rat and human using a novel data set of unbound drug concentrations in brain interstitial and cerebrospinal fluids. J. Med. Chem. 52, 6233–6243. 10.1021/jm901036q 19764786

[B27] Fu X. C. Wang G. P. Shan H. L. Liang W. Q. Gao J. Q. (2008). Predicting blood-brain barrier penetration from molecular weight and number of polar atoms. Eur. J. Pharm. Biopharm. 70, 462–466. 10.1016/j.ejpb.2008.05.005 18632257

[B28] Gao Z. Chen Y. Cai X. Xu R. (2017). Predict drug permeability to blood–brain-barrier from clinical phenotypes: Drug side effects and drug indications. Bioinformatics 33, 901–908. 10.1093/bioinformatics/btw713 27993785 PMC5860495

[B29] Geldenhuys W. J. Mohammad A. S. Adkins C. E. Lockman P. R. (2015). Molecular determinants of blood-brain barrier permeation. Ther. Deliv. 6, 961–971. 10.4155/tde.15.32 26305616 PMC4675962

[B30] Goldner J. A. (2012). Metoprolol-induced visual hallucinations: A case series. J. Med. Case Rep. 65, 1–3. 10.1186/1752-1947-6-65 PMC3295654 22336000

[B31] Goodwin J. T. Clark D. E. (2005). *In silico* predictions of blood-brain barrier penetration: Considerations to "keep in mind. J. Pharmacol. Exp. Ther. 315, 477–483. 10.1124/jpet.104.075705 15919767

[B32] Gratton J. A. Abraham M. H. Bradbury M. W. Chadha H. S. (1997). Molecular factors influencing drug transfer across the blood-brain barrier. J. Pharm. Pharmacol. 49, 1211–1216. 10.1111/j.2042-7158.1997.tb06072.x 9466345

[B33] Hemmateenejad B. Miri R. Safarpour M. A. Mehdipour A. R. (2006). Accurate prediction of the blood–brain partitioning of a large set of solutes using *ab initio* calculations and genetic neural network modeling. J. Comput. Chem. 27, 1125–1135. 10.1002/jcc.20437 16721721

[B34] Hou T. Xu X. (2002). ADME evaluation in drug discovery. 1. Applications of genetic algorithms to the prediction of blood-brain partitioning of a large set of drugs. J. Mol. Model. 8, 337–349. 10.1007/s00894-002-0101-1 12541001

[B35] Jiang L. Chen J. He Y. Zhang Y. Li G. (2016). A method to predict different mechanisms for blood–brain barrier permeability of CNS activity compounds in Chinese herbs using support vector machine. J. Bioinform. Comput. Biol. 14, 1650005. 10.1142/S0219720016500050 26632324

[B36] Kaliszan R. Markuszewski M. (1996). Brain/blood distribution described by a combination of partition coefficient and molecular mass. Int. J. Pharm. 145, 9–16. 10.1016/s0378-5173(96)04712-6

[B37] Kaznessis Y. N. Snow M. E. Blankley C. J. (2001). Prediction of blood-brain partitioning using Monte Carlo simulations of molecules in water. J. Comput. Aided. Mol. Des. 15, 697–708. 10.1023/a:1012240703377 11718475

[B38] Keserü G. M. Molnár L. (2001). High-throughput prediction of Blood−Brain partitioning: A thermodynamic approach. J. Chem. Inf. Comput. Sci. 41, 120–128. 10.1021/ci000043z 11206364

[B39] Kim T. You B. H. Han S. Shin H. C. Chung K.-C. Park H. (2021). Quantum artificial neural network approach to derive a highly predictive 3D-QSAR model for blood–brain barrier passage. Int. J. Mol. Sci. 22, 10995. 10.3390/ijms222010995 34681653 PMC8537149

[B40] Kola I. Landis J. (2004). Can the pharmaceutical industry reduce attrition rates? Nat. Rev. Drug Discov. 3, 711–715. 10.1038/nrd1470 15286737

[B41] Kortagere S. Chekmarev D. Welsh W. J. Ekins S. (2008). New predictive models for blood-brain barrier permeability of drug-like molecules. Pharm. Res. 25, 1836–1845. 10.1007/s11095-008-9584-5 18415049 PMC2803117

[B42] Kunwittaya S. Nantasenamat C. Treeratanapiboon L. Srisarin A. Isarankura-Na-Ayudhya C. Prachayasittikul V. (2013). Influence of logBB cut-off on the prediction of blood-brain barrier permeability. Biomed. Appl. Technol. J. 1, 16–34.

[B43] Lanevskij K. Japertas P. Didziapetris R. Petrauskas A. (2009). Ionization-specific prediction of blood–brain permeability. J. Pharm. Sci. 98, 122–134. 10.1002/jps.21405 18481317

[B44] Liu L. Zhang L. Feng H. Li S. Liu M. Zhao J. (2021). Prediction of the blood–brain barrier (BBB) permeability of chemicals based on machine-learning and ensemble methods. Chem. Res. Toxicol. 34, 1456–1467. 10.1021/acs.chemrestox.0c00343 34047182

[B45] Liu X. Tu M. Kelly R. S. Chen C. Smith B. J. (2004). Development of a computational approach to predict blood-brain barrier permeability. Drug Metab. Dispos. 32, 132–139. 10.1124/dmd.32.1.132 14709630

[B46] Lombardo F. Blake J. F. Curatolo W. J. (1996). Computation of brain-blood partitioning of organic solutes via free energy calculations. J. Med. Chem. 39, 4750–4755. 10.1021/jm960163r 8941388

[B47] Loryan I. Sinha V. Mackie C. Van Peer A. Drinkenburg W. H. Vermeulen A. (2015). Molecular properties determining unbound intracellular and extracellular brain exposure of CNS drug candidates. Mol. Pharm. 12, 520–532. 10.1021/mp5005965 25496026

[B48] Luco J. M. (1999). Prediction of the brain− blood distribution of a large set of drugs from structurally derived descriptors using partial least-squares (PLS) modeling. J. Chem. Inf. Comput. Sci. 39, 396–404. 10.1021/ci980411n 10192950

[B49] Ma X.-L. Chen C. Yang J. (2005). Predictive model of blood-brain barrier penetration of organic compounds. Acta Pharmacol. Sin. 26, 500–512. 10.1111/j.1745-7254.2005.00068.x 15780201

[B50] Mahringer A. Fricker G. (2016). ABC transporters at the blood–brain barrier. Expert Opin. Drug Metab. Toxicol. 12, 499–508. 10.1517/17425255.2016.1168804 26998936

[B51] Martins I. F. Teixeira A. L. Pinheiro L. Falcao A. O. (2012). A Bayesian approach to *in silico* blood-brain barrier penetration modeling. J. Chem. Inf. Model. 52, 1686–1697. 10.1021/ci300124c 22612593

[B52] Mcainsh J. Cruickshank J. M. (1990). Beta-blockers and central nervous system side effects. Pharmacol. Ther. 46, 163–197. 10.1016/0163-7258(90)90092-g 1969642

[B53] Miao R. Xia L.-Y. Chen H.-H. Huang H.-H. Liang Y. (2019). Improved classification of blood-brain-barrier drugs using deep learning. Sci. Rep. 9, 8802–8811. 10.1038/s41598-019-44773-4 31217424 PMC6584536

[B54] Muehlbacher M. Spitzer G. M. Liedl K. R. Kornhuber J. (2011). Qualitative prediction of blood-brain barrier permeability on a large and refined dataset. J. Comput. Aided. Mol. Des. 25, 1095–1106. 10.1007/s10822-011-9478-1 22109848 PMC3241963

[B55] Murakami H. Takanaga H. Matsuo H. Ohtani H. Sawada Y. (2000). Comparison of blood-brain barrier permeability in mice and rats using *in situ* brain perfusion technique. Am. J. Physiol. Heart Circ. Physiol. 279, H1022–H1028. 10.1152/ajpheart.2000.279.3.H1022 10993764

[B56] Narayanan R. Gunturi S. B. (2005). *In silico* ADME modelling: Prediction models for blood-brain barrier permeation using a systematic variable selection method. Bioorg. Med. Chem. 13, 3017–3028. 10.1016/j.bmc.2005.01.061 15781411

[B57] Norinder U. Sjöberg P. Österberg T. (1998). Theoretical calculation and prediction of brain–blood partitioning of organic solutes using MolSurf parametrization and PLS statistics. J. Pharm. Sci. 87, 952–959. 10.1021/js970439y 9687339

[B58] Obrezanova O. Csányi G. Gola J. M. R. Segall M. D. (2007). Gaussian processes: A method for automatic QSAR modeling of ADME properties. J. Chem. Inf. Model. 47, 1847–1857. 10.1021/ci7000633 17602549

[B59] Ooms F. Weber P. Carrupt P.-A. Testa B. (2002). A simple model to predict blood–brain barrier permeation from 3D molecular fields. Biochim. Biophys. Acta 1587, 118–125. 10.1016/s0925-4439(02)00074-1 12084453

[B60] Pardridge W. M. (2005). The blood-brain barrier: Bottleneck in brain drug development. NeuroRx 2, 3–14. 10.1602/neurorx.2.1.3 15717053 PMC539316

[B61] Platts J. A. Abraham M. H. Zhao Y. H. Hersey A. Ijaz L. Butina D. (2001). Correlation and prediction of a large blood-brain distribution data set--an LFER study. Eur. J. Med. Chem. 36, 719–730. 10.1016/s0223-5234(01)01269-7 11672881

[B62] Plisson F. Piggott A. M. (2019). Predicting blood–brain barrier permeability of marine-derived kinase inhibitors using ensemble classifiers reveals potential hits for neurodegenerative disorders. Mar. Drugs 17, 81. 10.3390/md17020081 PMC6410078 30699889

[B63] Radchenko E. V. Dyabina A. S. Palyulin V. A. (2020). Towards deep neural network models for the prediction of the blood–brain barrier permeability for diverse organic compounds. Molecules 25, 5901. 10.3390/molecules25245901 PMC7763607 33322142

[B64] Roberts G. Myatt G. J. Johnson W. P. Cross K. P. Blower P. E. (2000). LeadScope: Software for exploring large sets of screening data. J. Chem. Inf. Comput. Sci. 40, 1302–1314. 10.1021/ci0000631 11128088

[B65] Salminen T. Pulli A. Taskinen J. (1997). Relationship between immobilised artificial membrane chromatographic retention and the brain penetration of structurally diverse drugs. J. Pharm. Biomed. Anal. 15, 469–477. 10.1016/s0731-7085(96)01883-3 8953490

[B66] Sanchez-Covarrubias L. Slosky L. M. Thompson B. J. Davis T. P. Ronaldson P. T. (2014). Transporters at CNS barrier sites: Obstacles or opportunities for drug delivery? Curr. Pharm. Des. 20, 1422–1449. 10.2174/13816128113199990463 23789948 PMC3913737

[B67] Shah R. Babar A. Patel A. Dortonne R. Jordan J. (2020). Metoprolol-associated central nervous system complications. Cureus 12, e8236. 10.7759/cureus.8236 32582495 PMC7306637

[B68] Shen J. Du Y. Zhao Y. Liu G. Tang Y. (2008). *In silico* prediction of blood–brain partitioning using a chemometric method called genetic algorithm based variable selection. QSAR Comb. Sci. 27, 704–717. 10.1002/qsar.200710129

[B69] Shin H. K. Lee S. Oh H.-N. Yoo D. Park S. Kim W.-K. (2021). Development of blood brain barrier permeation prediction models for organic and inorganic biocidal active substances. Chemosphere 277, 130330. 10.1016/j.chemosphere.2021.130330 33780678

[B70] Silverman R. B. Lawton G. R. Ranaivo H. R. Chico L. K. Seo J. Watterson D. M. (2009). Effect of potential amine prodrugs of selective neuronal nitric oxide synthase inhibitors on blood–brain barrier penetration. Bioorg. Med. Chem. 17, 7593–7605. 10.1016/j.bmc.2009.08.065 19796958 PMC2775413

[B71] Singh M. Divakaran R. Konda L. S. K. Kristam R. (2020). A classification model for blood brain barrier penetration. J. Mol. Graph. Model. 96, 107516. 10.1016/j.jmgm.2019.107516 31940508

[B72] Soloway A. Whitman B. Messer J. (1960). Penetration of brain and brain tumor by aromatic compounds as a function of molecular substituents. J. Pharmacol. Exp. Ther. 129, 310–314. 13832708

[B73] Subramanian G. Kitchen D. B. (2003). Computational models to predict blood–brain barrier permeation and CNS activity. J. Comput. Aided. Mol. Des. 17, 643–664. 10.1023/b:jcam.0000017372.32162.37 15068364

[B74] Toropov A. A. Toropova A. P. Beeg M. Gobbi M. Salmona M. (2017). QSAR model for blood-brain barrier permeation. J. Pharmacol. Toxicol. Methods 88, 7–18. 10.1016/j.vascn.2017.04.014 28476566

[B75] Van De Waterbeemd H. Kansy M. (1992a). Hydrogen-bonding capacity and brain penetration. Chim. (Aarau). 46, 299–303. 10.2533/chimia.1992.299

[B76] Van De Waterbeemd H. Kansy M. (1992b). Hydrogen-bonding capacity and brain penetration. Chim. (Aarau). 46, 299–303. 10.2533/chimia.1992.299

[B77] Varadharajan S. Winiwarter S. Carlsson L. Engkvist O. Anantha A. Kogej T. (2015). Exploring *in silico* prediction of the unbound brain-to-plasma drug concentration ratio: Model validation, renewal, and interpretation. J. Pharm. Sci. 104, 1197–1206. 10.1002/jps.24301 25546343

[B78] Vilar S. Chakrabarti M. Costanzi S. (2010). Prediction of passive blood–brain partitioning: Straightforward and effective classification models based on *in silico* derived physicochemical descriptors. J. Mol. Graph. Model. 28, 899–903. 10.1016/j.jmgm.2010.03.010 20427217 PMC2873098

[B79] Wang W. Kim M. T. Sedykh A. Zhu H. (2015). Developing enhanced blood-brain barrier permeability models: Integrating external bio-assay data in QSAR modeling. Pharm. Res. 32, 3055–3065. 10.1007/s11095-015-1687-1 25862462 PMC4529363

[B80] Wang Z. Yang H. Wu Z. Wang T. Li W. Tang Y. (2018). *In silico* prediction of blood–brain barrier permeability of compounds by machine learning and resampling methods. ChemMedChem 13, 2189–2201. 10.1002/cmdc.201800533 30110511

[B81] Winkler D. A. Burden F. R. (2004). Modelling blood–brain barrier partitioning using Bayesian neural nets. J. Mol. Graph. Model. 22, 499–505. 10.1016/j.jmgm.2004.03.010 15182809

[B82] Wu Z. Xian Z. Ma W. Liu Q. Huang X. Xiong B. (2021). Artificial neural network approach for predicting blood brain barrier permeability based on a group contribution method. Comput. Methods Programs Biomed. 200, 105943. 10.1016/j.cmpb.2021.105943 33515846

[B83] Yan A. Liang H. Chong Y. Nie X. Yu C. (2013). *In-silico* prediction of blood-brain barrier permeability. Sar. QSAR Environ. Res. 24, 61–74. 10.1080/1062936X.2012.729224 23092117

[B84] Young R. C. Mitchell R. C. Brown T. H. Ganellin C. R. Griffiths R. Jones M. (1988). Development of a new physicochemical model for brain penetration and its application to the design of centrally acting H2 receptor histamine antagonists. J. Med. Chem. 31, 656–671. 10.1021/jm00398a028 2894467

[B85] Yuan Y. Zheng F. Zhan C.-G. (2018). Improved prediction of blood–brain barrier permeability through machine learning with combined use of molecular property-based descriptors and fingerprints. AAPS J. 20, 54–10. 10.1208/s12248-018-0215-8 29564576 PMC7737623

[B86] Zamek-Gliszczynski M. J. Chu X. Cook J. A. Custodio J. M. Galetin A. Giacomini K. M. (2018). ITC commentary on metformin clinical drug-drug interaction study design that enables an efficacy-and safety-based dose adjustment decision. Clin. Pharmacol. Ther. 104, 781–784. 10.1002/cpt.1082 29761830

[B87] Zhang D. Xiao J. Zhou N. Zheng M. Luo X. Jiang H. (2015). A genetic algorithm based support vector machine model for blood-brain barrier penetration prediction. Biomed. Res. Int. 2015, 292683. 10.1155/2015/292683 26504797 PMC4609370

[B88] Zhang L. Zhu H. Oprea T. I. Golbraikh A. Tropsha A. (2008). QSAR modeling of the blood-brain barrier permeability for diverse organic compounds. Pharm. Res. 25, 1902–1914. 10.1007/s11095-008-9609-0 18553217

[B89] Zhang Y.-Y. Liu H. Summerfield S. G. Luscombe C. N. Sahi J. (2016). Integrating *in silico* and *in vitro* approaches to predict drug accessibility to the central nervous system. Mol. Pharm. 13, 1540–1550. 10.1021/acs.molpharmaceut.6b00031 27015243

